# Mesenchymal Stromal Cell Treatment Alleviates Autism Spectrum Disorder Symptoms: A Case Report

**DOI:** 10.7759/cureus.83440

**Published:** 2025-05-04

**Authors:** Benjamin Gesundheit, Leah Hochbaum, Alexandra Fetyukhina, Vasily Vorobyev, Nikolay Vorobyev

**Affiliations:** 1 Research and Development, Cell El Ltd., Jerusalem, ISR; 2 Intensive Care Unit, Special Hospital for Internal Medicine, Belgrade, SRB; 3 General Medicine, Special Hospital for Internal Medicine, Belgrade, SRB

**Keywords:** asd, autism, case report, immunology, seizure, stem cell treatment

## Abstract

Autism spectrum disorder (ASD) is associated with significant lifelong challenges for severely affected children and their families. The condition remains poorly understood, and no reliable, effective treatments are available. Presented is a case of a boy with severe ASD, epilepsy, and a pathological electroencephalogram (EEG) who underwent five treatments between the ages of 5.75 years and nine years with mesenchymal stem cells from allogeneic placenta and umbilical cord tissue of unrelated donors. A significant clinical response was observed following each course, including the disappearance of seizures, normalization of the EEG after the first course, and continuously improved ASD symptoms, social skills, and emotional expression. Allogeneic mesenchymal stem cells might offer an attractive innovative modality for some children with ASD and may prove a promising therapy for children with seizure disorders. Clinical research directions are presented to develop these innovative treatments and design future research.

## Introduction

Autism spectrum disorder (ASD) affects one in 60 to 150 children, some of whom suffer from severely limited daily life functions, including deficits in communication, social interactions, and sensory perception, as well as repetitive and restrictive behaviors. Seizures affect ~20% of ASD children [1]. Given the wide range of manifestations, therapeutic options are vast, are symptom-directed, and must be individually tailored. These include pharmaceutical treatment to attenuate symptoms as well as highly structured and intensive behavioral, psychological, and/or educational therapy to build life skills and minimize challenging behaviors. However, many individuals fail to reach independence and require lifelong support.

Epidemiological, serological, immunogenetic, and radiological evidence increasingly point to immune and inflammatory etiology in a significant subset of children with ASD. Studies have linked hallmark ASD traits with dysfunctional innate and adaptive immune responses, maternal serum antibodies recognizing fetal brain antigens, elevated proinflammatory cytokine levels in the cerebrospinal fluid and plasma, and abnormal microglial size, density, and activity in the brain of children with ASD [2-7].

While targeted biological therapies do not exist, mesenchymal stromal cell (MSC)-based therapy (MSCT) has been proposed due to its strong immune-modulating effects across human leukocyte antigen (HLA) barriers [8]. This unique non-hematopoietic, multipotent cell population bears notable immunomodulatory, homing, and secretory capacities and has been safely integrated into a wide range of clinical applications, including treatment of refractory graft vs. host disease, chronic inflammatory disorders, heart diseases, and a range of autoimmune diseases [9]. We present the remarkable improvement of a child with severe ASD and seizures who underwent a series of five MSCTs. Insights gained from this innovative therapeutic approach are proposed for future research.

## Case presentation

A 32-month-old boy was diagnosed with ASD (Autism Diagnostic Observation Schedule (ADOS) score: 23). At the time of diagnosis, the child showed poor coordination, tired after walking ~50 m, suffered from tremors and dyskinesia, and was oblivious to his surroundings and minimally communicative. Unexplained regression of his typical developmental course had begun at the age of 18 months, with gradual loss of all acquired verbal and communication skills.

**Figure 1:**
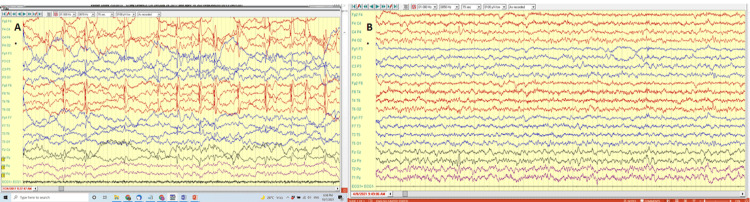
EEG findings before and after alloMSCT#1 A) Pre-treatment sleep electroencephalogram recording showing epileptiform discharges, and multi-spikes over the left hemisphere with secondary generalization (July 24, 2017). B) Post-treatment EEG showing normal sleep recording with no epileptiform discharges (August, 2021). EEG: electroencephalogram; alloMSCT#1: allogeneic mesenchymal stromal cell-based therapy number 1

Occupational and communication therapies were recommended and immediately implemented. Karyotypic analysis identified a duplication on chromosome 4q16.3. Sequence analysis identified intronic variations in the SLC13A5 and CACNA1H genes, with the former associated with autosomal recessive early infantile epileptic encephalopathy type 25 and the latter with autosomal dominant generalized epilepsy syndromes. Fragile X syndrome was ruled out. The child had allergic reactions to mosquito bites and suffered since birth from severe reflux with daily vomiting and associated heartburn, resulting in poor weight gain. One month after diagnosis, he had tonic-clonic seizures, with electroencephalogram (EEG) recordings showing high-frequency centro-temporal spikes in the left hemisphere without generalizations (Figure 1A). Anticonvulsive valproate (Depalept) treatment (≤350 mg x2/d) was initiated, but due to poor seizure control, carbamazepine (Trileptal) (150 mg twice daily (BID)), levetiracetam (Keppra) (500 mg BID), Avidekel cannabidiol oil (seven drops three times daily (TID)), and clonazepam (Klonopin) (four drops) was added as rescue medications for prolonged seizure. However, the child was still hospitalized with uncontrolled seizures eight times within one year. He suffered severe weight loss due to poor food intake and uncontrolled, prolonged, and frequent seizures with life-threatening severe apnea. The mother suffered hypothyroidism during pregnancy with the child, which was treated with Eltroxin. No other pregnancy complications were reported. The child was born at full term.

Family history

The child had an 11-month-old brother with epilepsy and typical early signs of autism and a seven-year-old normally developed sister. Both parents suffered from asthma, attention-deficit/hyperactivity disorder (ADHD), and learning difficulties, and the father from hypothyroidism. Both functioned normally as parents and professionally. The mother had an extensive family history of learning difficulties and ADHD, including one uncle with mild autism and with a daughter diagnosed with ASD. Another uncle suffered from Crohn’s disease and had one daughter with ASD and another with bipolar disorder. The father had a family history that included hypothyroidism and one brother with ASD and significant social anxiety. 

Allogeneic mesenchymal stromal cell-based therapy

The parents provided informed consent for the child to undergo intravenous allogeneic MSCTs (alloMSCT) from unrelated mothers and babies (Table 1; Appendices A, B). Throughout the treatment period, the child continued with the same schedule of occupational and communication therapies. All assessments were performed and documented at the Institute for Child Development, affiliated with the child’s state-mandated health fund, by independent pediatricians specializing in neurology and/or child development. At age five years and eight months, the child received one dose of 2.5x10^6^ placenta-derived (PD) MSC/kg (MSCT#1; March 2019). Two days later, the child was more relaxed and started to play with his siblings. No seizures were experienced, and EEG evaluation 18 weeks after treatment was normal (Figure 1B). In parallel, the child began to communicate with longer sentences and better pronunciation, initiated conversations, and showed social interest and interaction. He was weaned off baby bottles and nappies, reflecting developmental maturation. Furthermore, vomiting frequency and heartburn intensity improved, resulting in better appetite and weight gain.

**Table 1:** Details of the patient's treatment course alloPD-MSC: allogenic cultivated placenta-derived multipotent mesenchymal stroma cells, administered intravenously; alloUC-MSC: allogenic cultivated umbilical cord-derived multipotent mesenchymal stromal cells, administered intravenously; d: days

Parameters	Mesenchymal stromal cell treatments				
	#1 (10-13 March 2019)	#2 (28 July-2 August 2019)	#3 (1-7 March 2020)	#4 (25-29 July 2021)	#5 (9-14 August 2022)
Age (years (y)/months (mo) and weight (kg))	5 y + 8 mo (16 kg)	6 y + 0.75 mo (18.5 kg)	6 y + 8 mo (19.5 kg)	8 y (23.5 kg)	9 y (26.5 kg)
alloPD-MSC (million/kg)	2.5 (IV)	2.2 (IV)	2.1 (IV)	1.7 (IV)	Day 1: 1.5 (IV) Day 2: 1.5 (IV)
					Days 1 and 2 IM: 1.5x10^6 ^cells
alloUC-MSC (million/kg)		2.2 (IV)	3.1 (IV)	1.7 (IV)	Day 1: 1.5 (IV) Day 2: 1.5 (IV)
					Days 1 and 2 IM: 1.5x10^6 ^cells
Exosomes			Nasal spray x2 in 5 d	Nasal spray x3 in 5 d	Nasal spray x2 5 d + x2/d for 30 d

The MSCT#2 (2.2x10^6^ PD-MSC/kg) dose was administered four months later, followed two days later by 2.2x10^6^ MSC/kg umbilical cord-derived MSC (UCD-MSC). This led to significant sensorimotor improvements, as manifested by the ability to walk for 15 to 20 minutes without tiring and sufficiently coordinated movements to swing independently. All anticonvulsive medications and cannabis were gradually discontinued. By the end of the 12-week treatment withdrawal (December 2019), he began to hold a pencil and was able to draw simple stick figures and letters, which he had never been able to do given tremors and dyskinesia. Speech and social interaction improved, as exhibited through awareness of surroundings, expanded vocabulary, and spontaneous sentences, albeit still short and very structured (January 2020).

For MSCT#3, delivered eight months later (March 2020), 2.1x10^6^ PD-MSC/kg were intravenously applied, followed two days later by an infusion of 3.1x10^6^ alloUCD-MSC/kg. Exosomes produced from the same number of MSCs of the same donors (3x10^6^ MSC/kg, resulting in an equivalent of 4x10^8^ units of exosomes) were intranasally delivered on the days of PD- and UC-MSC treatment. The child experienced a short episode of fever and shivering, which resolved after administration of paracetamol. Follow-up evaluations when the child was seven years old showed significant improvements in emotional development. For the first time, the child began to express his feelings and clearly describe physical discomforts, initiate conversations with strangers, greet people, read full storybooks aloud in English, speak in Hebrew, and invent games with his own rules.

A full workup was performed at age 7.5 years to assess heartburn and vomiting. Eosinophilic esophagitis was treated with Nexium gel and topical budesonide (0.5 mg x 2 d/d).

Due to travel restrictions during the coronavirus pandemic, MSCT#4 was delayed to 18 months after MSCT#3. The dose administered was reduced due to the mild side effects experienced in the previous treatment session. The regimen included an IV infusion of 1.7x10^6^ PD-MSC/kg, followed two days later by an IV infusion of 1.7x10^6^ UC-MSC/kg. In parallel, exosomes were administered intranasally once daily for three consecutive days, starting from the day of PD-MSC administration. Over the first four to six weeks after MSCT#4, less anxiety and hyperactivity were observed, with further improvements in speech, manifested by the use of more words, longer sentences, and better pronunciation. The child connected, communicated, engaged more with friends, played with both older and younger children, was significantly less aggressive and more attentive to others’ needs, and showed further improvements in expressing emotions, including use of facial expressions and other forms of non-verbal communication. He also exhibited more independence in daily activities (shower, homework, etc.). The child could understand complex instructions, including planning, and was able to execute plans without being reminded. Teachers reported improved attention, reading and writing, more academic interest, and better reactions to challenging social situations.

The MCST#5 dose was delivered 11 months later (August 2022) and involved two consecutive days of treatment with intravenous infusion of UC-MSC and PD-MSC (1.5x10^6 ^cells/kg each), intramuscular injection of both cell types (1.5x10^6^ cells each) and intranasal MSC exosomes. Intranasal exosome treatment (one to two drops) was continued twice daily for 30 days. In the subsequent six months, the child’s parents reported significant cognitive improvements, which included faster and more crystallized thinking skills, more mature and rational decision-making, and improved expression of emotional and physical needs and desires, including the yearning for social interactions with peers. In addition, he showed more willingness to execute chores. The child’s improvements were reflected in all subdomains of the Autism Treatment Evaluation Checklist (ATEC) [10] and Social Responsiveness Scale-2 (SRS second edition) [11] scales, with scores moving into the normal range. Of note, standard blood work (complete blood count, thyroid and liver functions, biochemistry, and chemistry panels) performed before each treatment session showed values all within the normal range.

## Discussion

The child with severe ASD, intractable seizures, and gastrointestinal symptoms showed continuous positive clinical responses to five alloMSCTs (Table 2). Improvements included reduced classical features of autistic behavior, resolution of seizures and gastrointestinal symptoms, improved social skills and emotional expression, and normalization of pathological EEG.

**Table 2:** Clinical improvements in the patient as measured by two psychological tests and parental/teacher’s observations The Autism Treatment Evaluation Checklist (ATEC) is comprised of four subscales: I. Speech/language communication (14 items), II. Sociability (20 items), III. Sensory/cognitive awareness (18 items), and IV. Health/physical/behavior (25 items). The lower the score, the less severe autism manifestations; The Social Responsiveness Scale Edition 2 (SRS2), a 65-item scale, is comprised of subscales: I. Social awareness (eight items), II. Social cognition (12 items), III. Social communication (22 items), IV. Social motivation (11 items), and V. Restricted interests and repetitive behavior (12 items). Each item was scored on a four-point Likert-type scale. tx: treatment

Treatment/Evaluation day		Pre-tx #1 10/03/2019	Pre-tx #2 25/07/2019	Pre-tx #3 02/03/2020	Pre-tx #4 25/07/2021	One month post tx #4	Six months post-Tx #5
Age (years (y) and months (mo))		5 y, 8 mo	6 y, 0.75 mo	6 y, 8 mo	8 y, 0.75 mo	8 y, 1.75 mo	9 y, 7 mo
ATEC [10]	Speech and language	24	12	8	5	4	2
	Sociability	32	23	16	11	8	4
	Sensory/Cognitive awareness	29	15	7	1	2	0
	Health/Physical behavior	64	54	41	31	11	5
	Total score	149	104	72	48	25	11
SRS2 [11]	Awareness	23	13	12	8	8	0
	Cognition	36	29	25	17	19	4
	Communication	65	48	38	23	25	15
	Motivation	30	28	17	10	12	7
	Repetitive behaviors	39	33	26	20	7	6
	Total score	193	151	118	78	71	32
Parental/Teacher's observations		Poor coordination, tired after walking; ~50m tremors; dyskinesia; oblivious to surroundings; minimally communicative	More relaxed; plays with siblings; no seizures; normal EEG; communicates with longer sentences, better pronunciation, and initiation of conversation; social interest and interaction; weaned off bottles and nappies.	Walk 15-20 min without tiring; coordinated movements like swinging independently; holding a pencil; drawing stick figures and letters; awareness of surroundings; expanded vocabulary; spontaneous sentences (short and structured).	Expression of feelings; initiates conversation with strangers; greets people; Reading full storybooks in English; speaking Hebrew; inventing games	Reduced anxiety and hyperactivity; expanded vocabulary; longer sentences; improved pronunciation; increased communication; engagement with friends; less aggressive and more attentive; improved expression of feelings, including nonverbal communication; increased independence; improved understanding of complex instructions	Significant cognitive improvement with faster and more crystallized thinking; more mature and rational decision-making; improved expression of emotional and physical needs; increased willingness to perform chores.

Below are points for consideration in future research.

This child’s extensive family history of autoimmune diseases, ASD, and other neurological-psychiatric conditions aligned with previous reports of an association between ASD and the immune system [12]. This suggests an immunological basis for a subgroup of ASD, with the potential to diagnose and monitor treatment response by autism-specific biomarkers [13].

The immediate disappearance of the child’s severe seizures with stable and lasting normalization of his EEG traces and consecutive discontinuation of all anticonvulsive medication with simultaneous clinical improvement of ASD symptoms provide evidence of the established link between epilepsy and ASD [14]. While many researchers exclude ASD patients with epileptic manifestations from MSCT, its therapeutic benefit for epilepsy [15] suggests that seizures should not be a contraindication for MSCT. Furthermore, epileptic EEG findings may serve as an objective tool to assess and monitor the therapeutic success of MSCT. 

Gastrointestinal symptoms, including eosinophilic esophagitis, are a common comorbidity of ASD [16]. In their multicenter phenotypic characterization of 705 patients with eosinophilic esophagitis, Chehade et al. report that up to 30% of patients with the disease had a neurodevelopmental disorder [17], with the gut-brain axis being a common denominator. The clinical improvement after MSCT#1 was remarkable. 

Stem cell biology is a new field with promising potential for many clinical conditions, including neurological, neurodegenerative, or immunological disorders and stroke [8]. While autologous cord blood is available for some children, low MSC content and reduced activity after years of cryopreservation limit its utility. Therefore, PD- and UCD-MSCs from healthy donors might be useful since these organs protect the embryo from the mother’s immune system and can be collected fresh [8]. Sun et al. reported that 50% of their 12-patient ASD cohort aged two and 11 years, intravenously administered up to three doses of UCD-MSCs (2x10^6^ cells/kg) from unrelated donors showed improvements in at least two of three ASD-specific measures [18]. The measures included social communication skills, autism symptom severity, and expert clinical assessment and were evaluated six months after the last treatment dose. Anti-HLA antibodies were detected in one out of three of the treated children, none of whom exhibited clinical sequelae. Similarly, a single infusion of a median dose of 2.6 x 107 autologous UCD-MSC/kg to 25 children with ASD led to improvements in core ASD symptoms in approximately 60% of the participants [19]. A follow-up analysis identified associations between changes in brain networks and behavioral and communication improvements [20]. The same team later reported significant improvements in attention and communication skills among a subgroup of children with ASD but without intellectual deficit within six months of treatment with a single infusion of autologous or allogeneic UCD-MSC [21]. Due to the distinct secreted cytokine and growth factor profiles of MSCs from different anatomical regions [22], umbilical cord-derived MSCTs were added after session 1 once the clinical tolerability of the placenta-derived cell treatment was proven.

The optimal age and regimen for MSCT remain to be determined. Administration at an early age, when brain plasticity is still high, is expected to be more effective. Since psychological tests are reliable only from the age of ±3 years, ASD diagnosis limits administration for very young children. Therefore, besides optimizing treatment, objective ASD diagnostic tools suitable for children under three years of age are critical [13]. In parallel, dosing intervals and the number of treatments need optimization. Furthermore, the standard dose for IV administration of MSCs to children is 3x10^6^ cells/kg body weight, with 0.5x10^6^ cells/kg body weight reported as the minimum effective dose. While MSC doses of up to 10.7x10^6^ cells/kg were well tolerated, the effective therapeutic range seems broad and will require calibration [19]. In the current case, the child’s MSCT doses were gradually intensified to optimize their effect based on the tolerance of the previous dose. 

Despite accumulating evidence regarding the potential of cell therapy strategies for the treatment of ASD symptoms, the underlying molecular mechanisms remain elusive. The suggested paracrine effect of MSC-secreted exosomes rich in RNA and protein with immunomodulatory and restorative potential opens promising options for exosomes in cell-free therapies or as adjunct therapeutic agents [23, 24]. In our patient, it was unclear if the addition of exosomes to the MSC#3-MSC#5 treatments provided any added clinical benefit. Therefore, no definitive recommendations regarding their inclusion in future protocols can be made based on the present case.

## Conclusions

The presented case demonstrated remarkable improvements in social, verbal, and cognitive skills and full resolution of seizure and gastrointestinal symptoms in a child with severe autism and intractable seizures treated with a five-session allogeneic stem cell transplantation program. The case supports the mounting interest in stem cell therapy for children with autism and warrants further evaluations of this avenue as a therapeutic modality.

## Appendices

Appendix A

Mesenchymal Stromal Cell Isolation and Processing

The placenta and umbilical cord were obtained from a healthy donor during delivery. Within 6-12 hours, the material was delivered to the laboratory in an airtight container with DPBS + 2% AB/AM at +4°C. The cells were isolated using the enzymatic method and the explant method. Thereafter, the cells were cultured in Xeno Free medium (DMEM/F12 + AB/AM 1% MesenCult supplement). After two to three passages, the cells were collected and cryopreserved, and samples were later analyzed for compliance with MSC treatment standards (phenotyping, karyotyping, confirmation of differentiation potential) and for sterility (fungal, viral (hepatitis B, hepatitis C, HIV, herpes, CMV), toxoplasma, and mycoplasma infections) by PCR.

Before administration to the patient, the cells were thawed, purified from cryopreservant, and cultured in a Xeno-Free medium (DMEM/F12 + AB/AM 1%) until they reached the lag phase of growth and confluence of 75-80%. The cells are checked again for sterility and the absence of endotoxins. On the day of administration, the cells were collected and rinsed to remove all nutrients, antibiotics, and other reagents that were in the medium and counted, and viability was determined (at least 90%). The cells were injected into the infusion solution (0.9% NaCl) and administered to the patient over a maximum of 60 minutes.

Appendix B

Exosome Preparation

Exosomes were obtained from umbilical cord MSCs no older than three passages, collected as described above. The required number of cells were collected, rinsed, transferred to DPBS, and conditioned under strictly specified conditions within 24 hours. Thereafter, samples were filtered, concentrated, and subjected to ultradiafiltration. The finished product was stored in a strictly specified concentration at -80°C.
